# Supplementary figures and images for: PIG-1 MELK-dependent phosphorylation of nonmuscle myosin II promotes apoptosis through CES-1 Snail partitioning
Source: PLoS Genet. 2020 Sep 18;16(9):e1008912. doi: 10.1371/journal.pgen.1008912 (PMC7527206; doi:10.1371/journal.pgen.1008912)

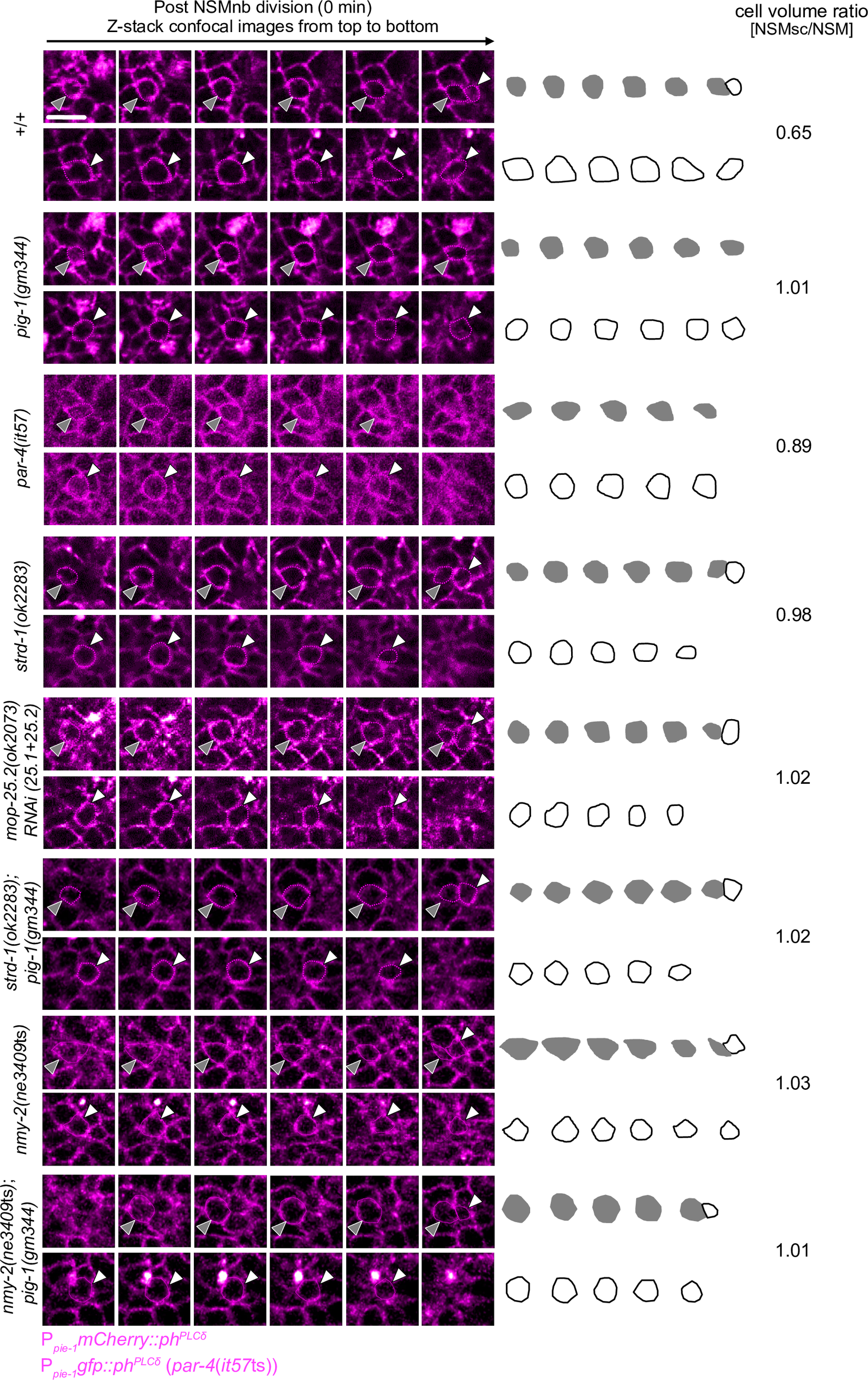

Supplement: S1 Fig — (Left) Series (Z-stacks from top to bottom) of fluorescence confocal images of the NSM and NSMsc immediately after NSMnb division in wild-type and mutant embryos expressing the transgene Ppie-1gfp::phPLCδ (bcIs57) (par-4(it57ts) mutant background) or Ppie-1mCherry::phPLCδ (ltIs44) (all the other mutant backgrounds), which expresses a fusion protein that labels the cell boundary (magenta). Grey and white arrow heads point to the NSMsc or NSM, respectively. (Right) Schematic representations of the areas of the NSM (white) or NSMsc (grey) for each genotype and corresponding cell volume ratios [NSMsc/NSM]. Scale bar 5 μm. (TIF) [file pgen.1008912.s001.tif]

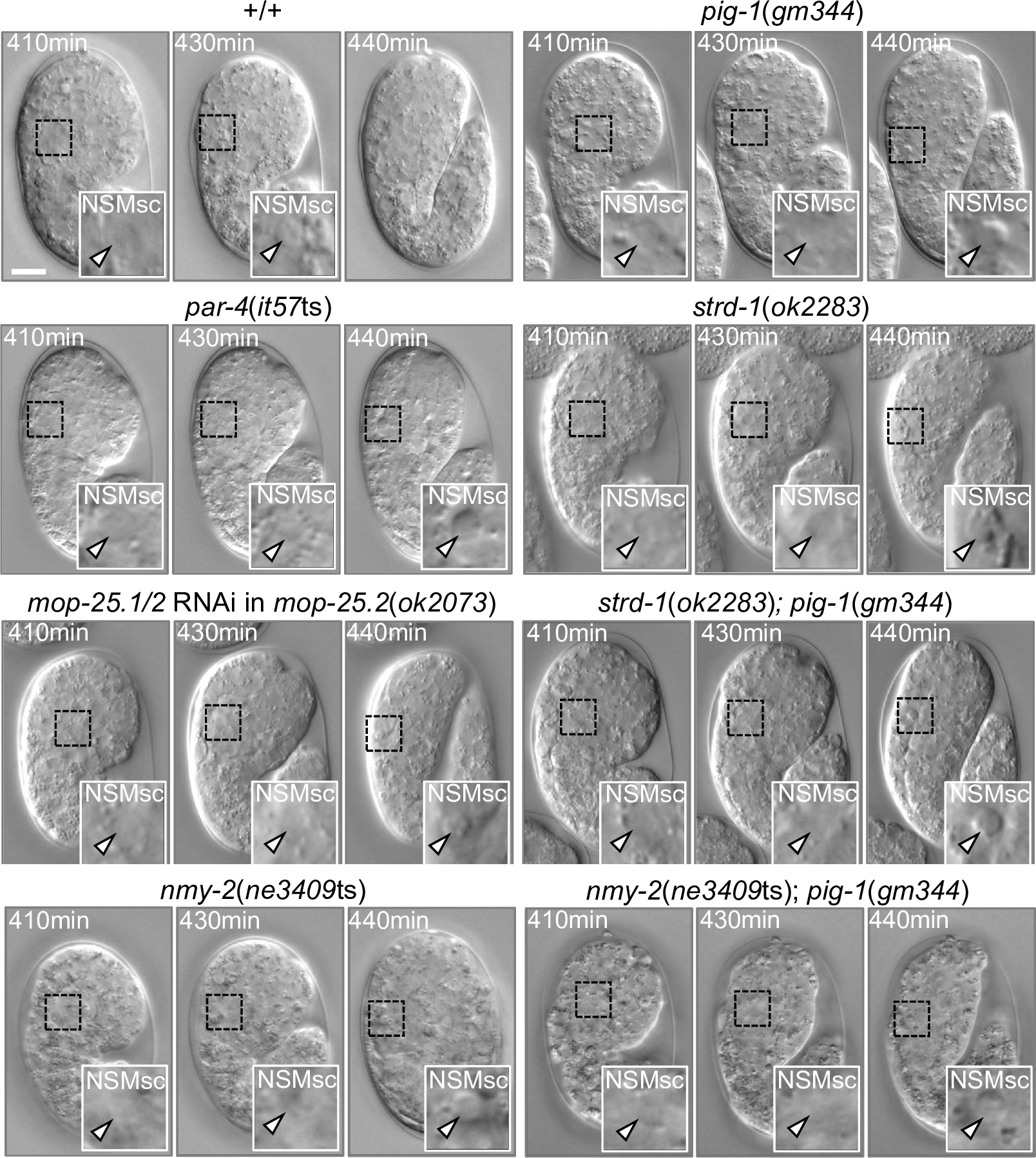

Supplement: S2 Fig — Nomarski images of representative wild-type and mutant embryos at different times post fertilization [min]. 410 min is immediately post NSMnb cytokinesis. Insets show the NSMsc and white arrow heads point to relevant cells. Scale bar 10 μm. (TIF) [file pgen.1008912.s002.tif]

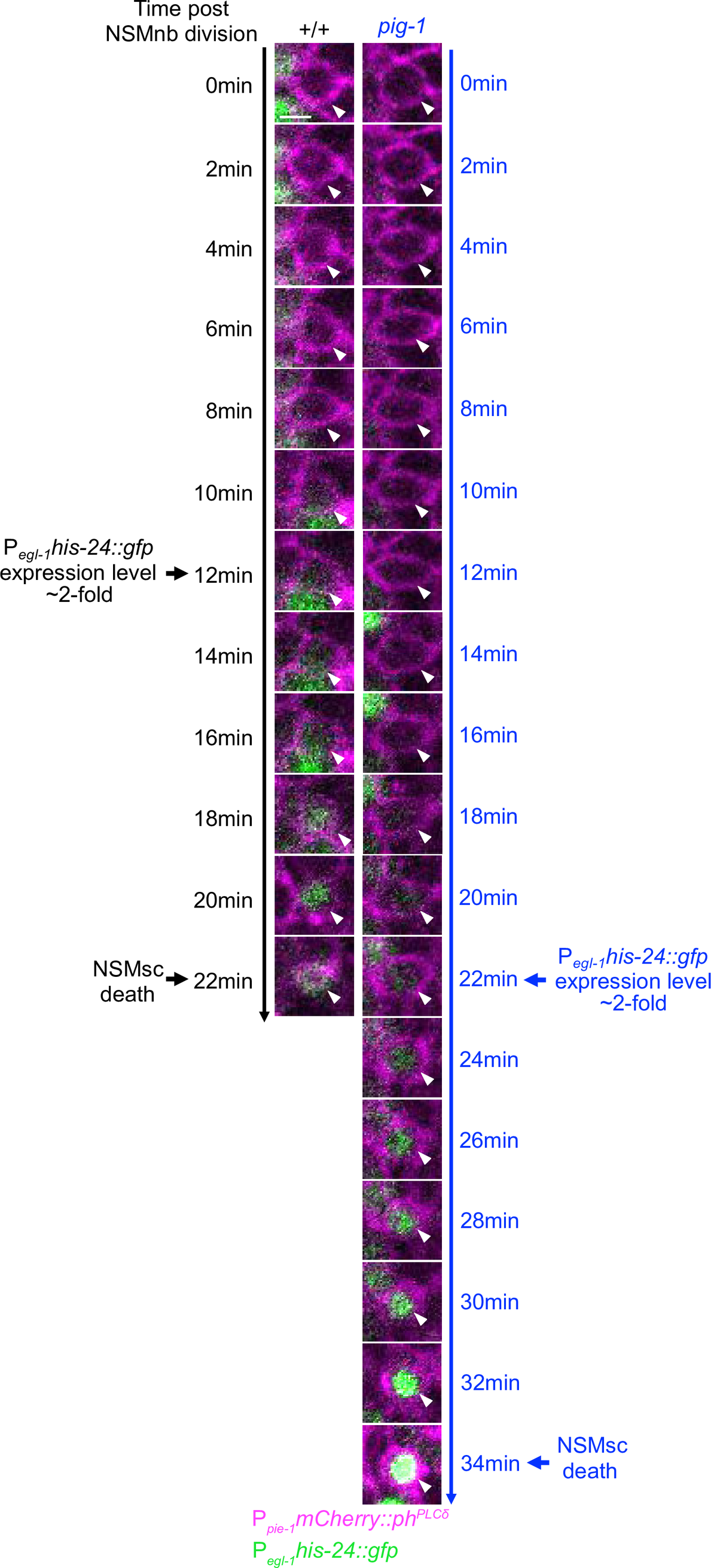

Supplement: S3 Fig — Fluorescence images of NSMsc in representative wild-type [+/+] and pig-1(gm344) embryo carrying the transgenes Pegl-1his-24::gfp (bcIs37) (green) at different time points post NSMnb division [min]. Cell boundaries are labeled with transgene Ppie-1mCherry::phPLCδ (ltIs44) (magenta). White arrow heads point to NSMsc. Scale bar 2 μm. (TIF) [file pgen.1008912.s003.tif]

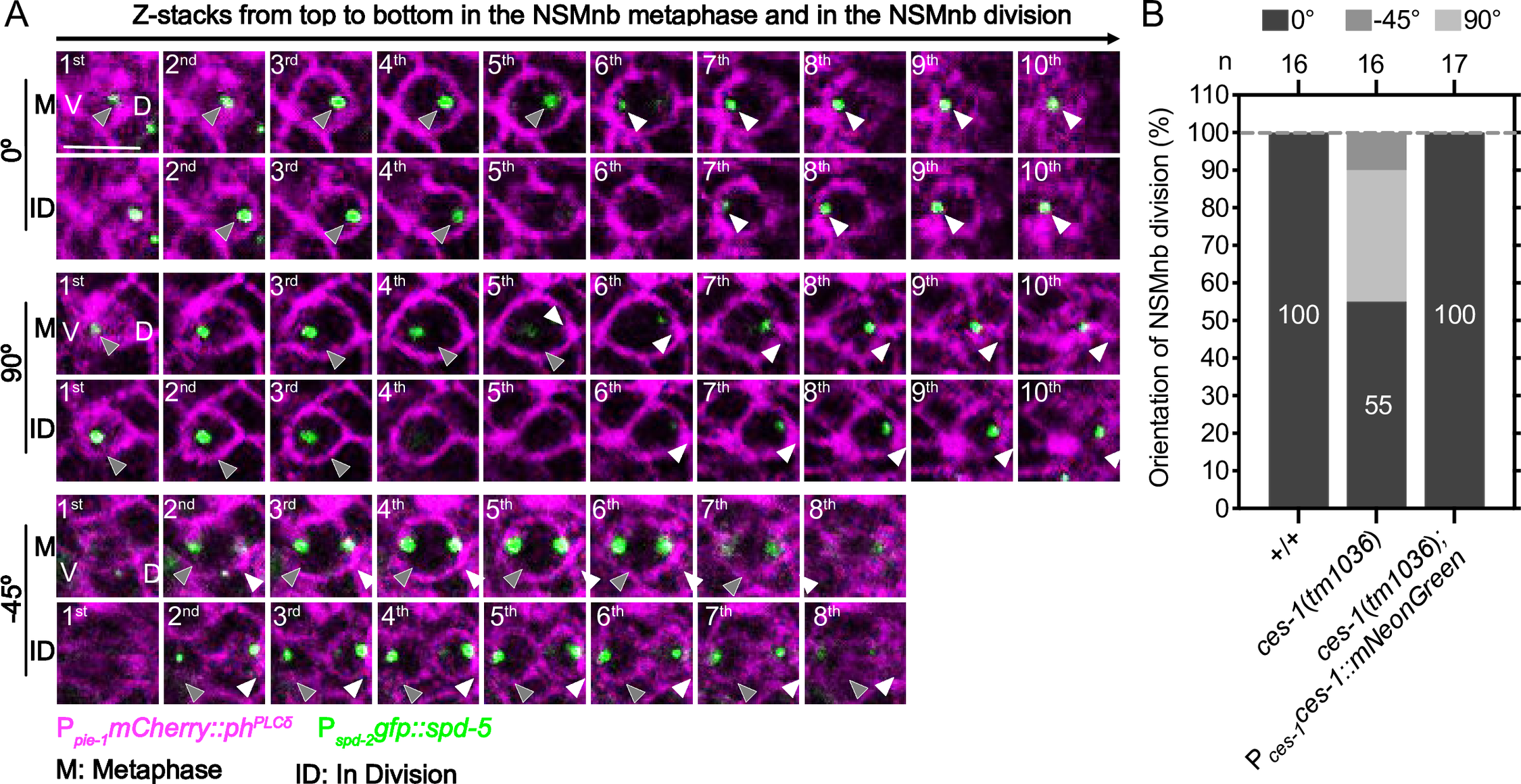

Supplement: S4 Fig — (A) Series (Z-stacks from top to bottom) of fluorescence confocal images of three different ces-1(tm1036) embryos (0°, 90°, -45°) at NSMnb metaphase (M) and in NSMnb division (ID). All embryos were homozygous for transgene Pspd-2::gfp::spd-5(ltSi202) (green), which labels the centrosomes, and for transgene Ppie-1mCherry::phPLCδ (ltIs44) (magenta), which labels cell boundaries. Grey arrow heads point to the centrosomes, which are segregated to the NSMsc, and white arrow heads point to the centrosomes, which are segregated into the NSM post NSMnb division. 0° indicates the ‘wild-type’ orientation of the NSMnb cleavage plane. 90° and -45° indicate ‘mutant’ orientations of the NSMnb cleavage plane found in ces-1(tm1036) mutants. (B) Quantification of the fraction of different orientations of the NSMnb cleavage plane in embryos of various genotypes (n = 16–17). Transgene Pces-1ces-1::mNeonGreen (bcSi66) fully rescues the defect in the orientation of the NSMnb cleavage plane observed in ces-1(tm1036). (TIF) [file pgen.1008912.s004.tif]

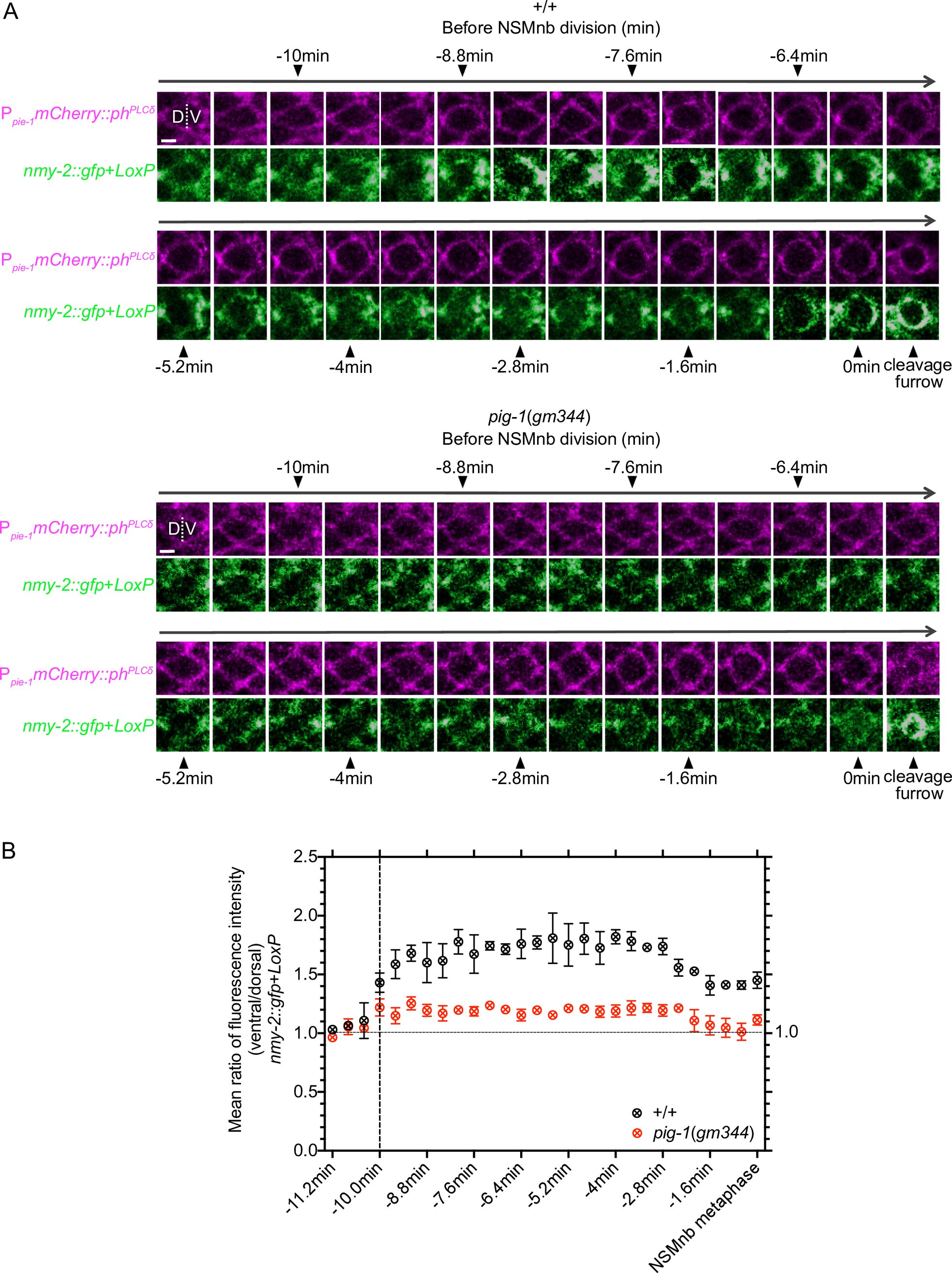

Supplement: S5 Fig — (A) Series of fluorescence confocal images of nmy-2::gfp+LoxP (cp13) (green) in representative NSMnb in wild-type [+/+] and pig-1(gm344) embryo. Recordings were performed from ~ -11 min prior to NSMnb division until the NSMnb divided (0 min). Cell boundaries are labeled with transgene Ppie-1mCherry::phPLCδ (ltIs44) (magenta). D indicates dorsal and V ventral. White dotted line indicates the middle of the NSMnb. Scale bar 2 μm. (B) Shown are mean ratios of fluorescence intensities + SEM [Pnmy-2nmy-2::gfp] in the ventral to dorsal half of the NSMnb at different times prior to NSMnb division are shown (n = 3). Vertical red dotted indicates the time point at which asymmetry of NMY-2::GFP in wild type was established. For each genotype, the mean ratios obtained were normalized to the mean ratio at recording time -11.2 min, which was set to 1. A ratio of 1.0 is indicated by the horizontal black dotted line. (TIF) [file pgen.1008912.s005.tif]

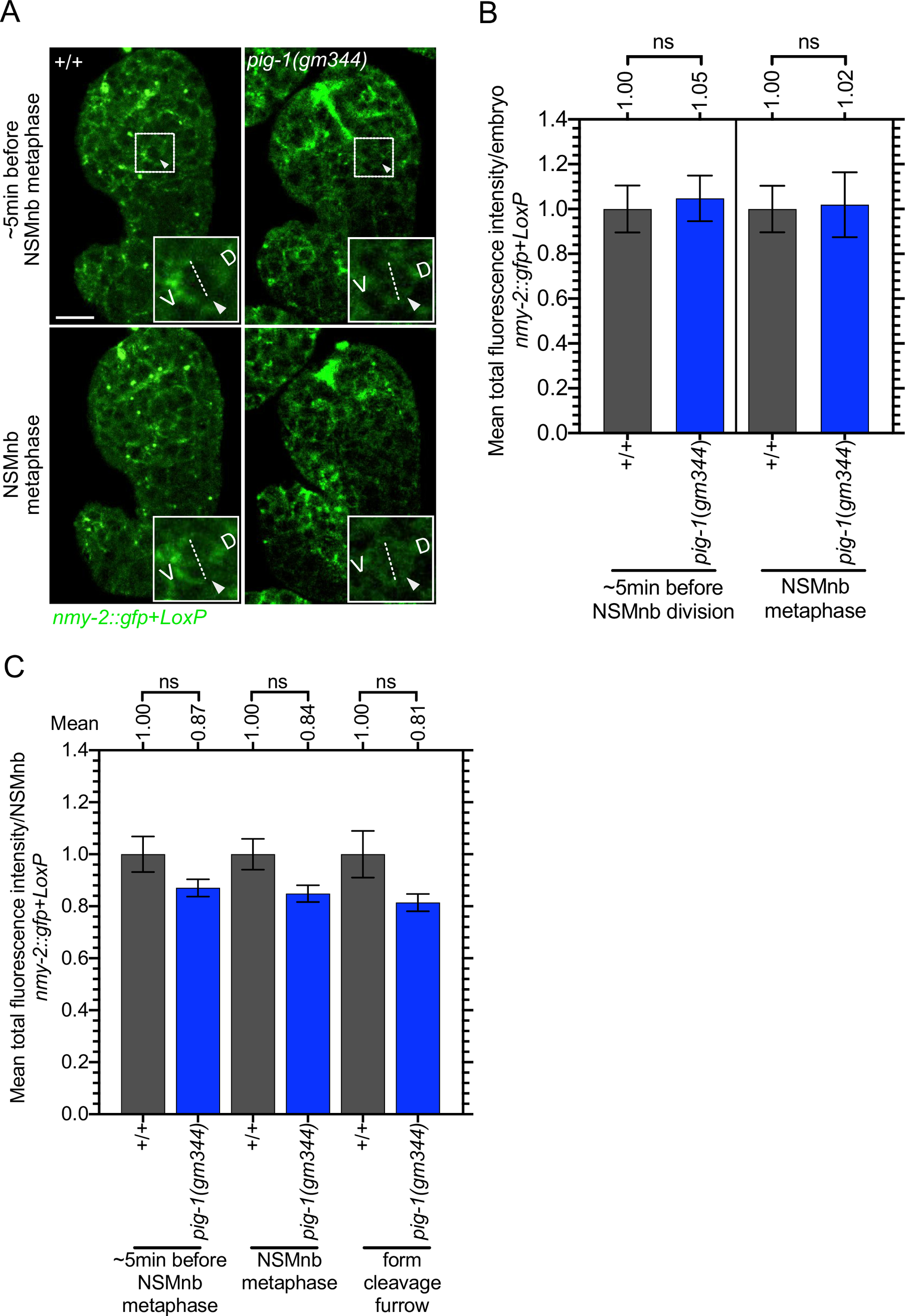

Supplement: S6 Fig — (A) Fluorescence confocal images of nmy-2::gfp+LoxP (cp13) in representative wild-type [+/+] and pig-1(gm344) embryo ~5 min before the NSMnb metaphase and at NSMnb metaphase. Insets show the NSMnb and white arrow heads point to the NSMnb, D and V indicate dorsal and ventral half of the NSMnb. White dotted lines indicate middle of NSMnb. Scale bar 10 μm. (B) Quantification of NMY-2::GFP in embryos. Shown are mean total fluorescence intensities per embryo in wild type [+/+] and pig-1(gm344) ~5 min before NSMnb metaphase and at NSMnb metaphase (n = 12–14). All embryos analyzed were homozygous for the transgene nmy-2::gfp+LoxP(cp13) (green). Statistical significance was determined using Mann–Whitney test (ns, no significance). (C) Quantification of NMY-2::GFP in NSMnb. Shown are mean total fluorescence intensities per NSMnb in wild type [+/+] and pig-1(gm344) ~5 min before NSMnb metaphase, at NSMnb metaphase and at the time the cleavage furrow forms (n = 12–14). All embryos analyzed were homozygous for the transgene nmy-2::gfp+LoxP(cp13) (green). Statistical significance was determined using Mann–Whitney test (ns, no significance). (TIF) [file pgen.1008912.s006.tif]

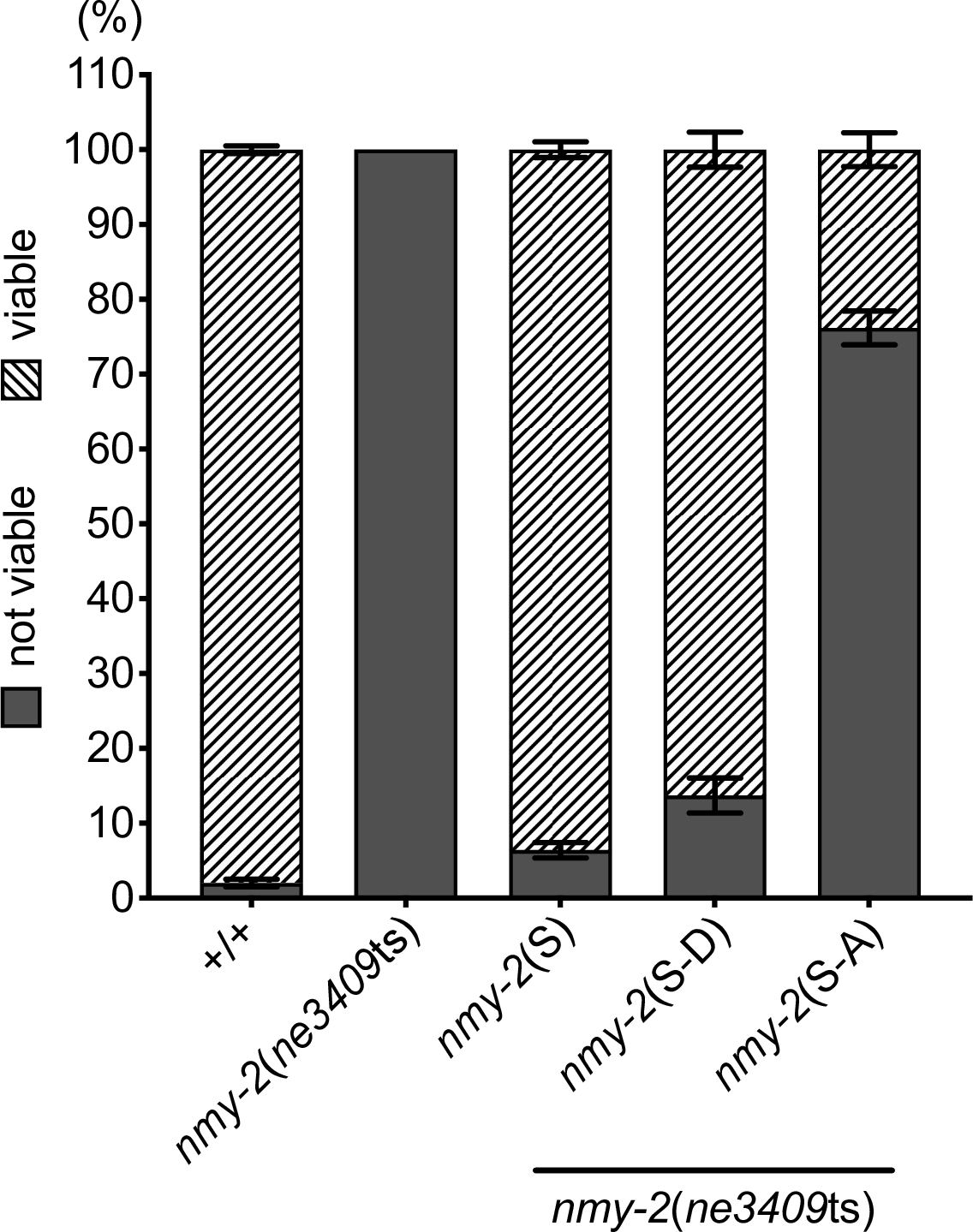

Supplement: S7 Fig — Embryonic viability [%] at 25°C in different genotypes. nmy-2(S), nmy-2(S-D), nmy-2(S-A) represent single-copy transgenes Pnmy-2nmy-2 (bcSi97), Pnmy-2nmy-2S211DS1974D (bcSi102) and Pnmy-2nmy-2S211AS1974A (bcSi101), respectively. For each genotype, three independent experiments were performed (n = 240–290). Date are presented as mean ± SEM. (TIF) [file pgen.1008912.s007.tif]

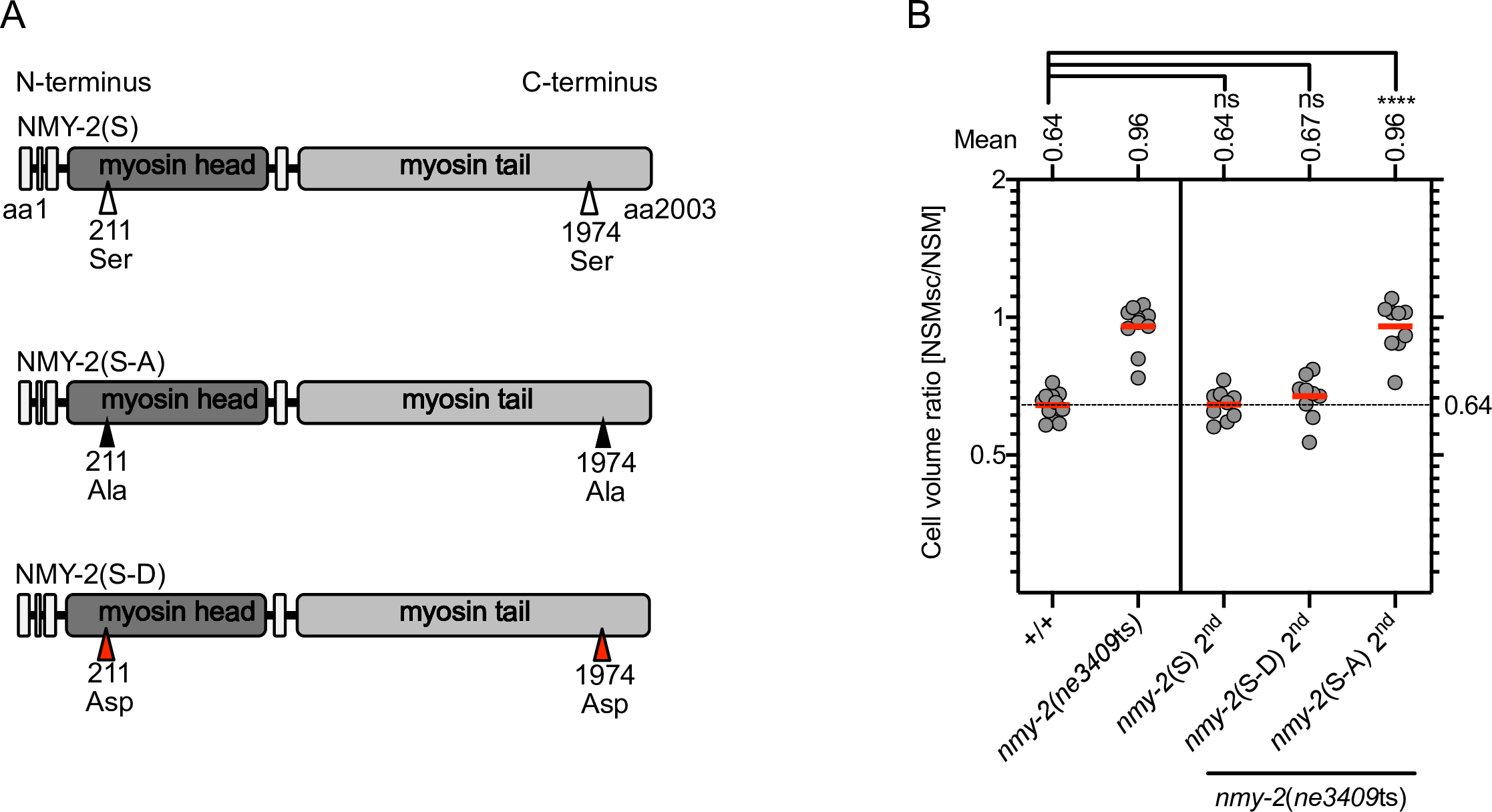

Supplement: S8 Fig — (A) Schematic representations of wild-type and mutant NMY-2 proteins. Mutant protein NMY-2(S-A) cannot be phosphorylated at S211 and S1974. Mutant protein NMY-2(S-D) mimics wild-type NMY-2 protein phosphorylated at S211 and S1974. Wild-type NMY-2 protein is referred to as NMY-2(S) (B) Cell volume ratio [NSMsc/NSM] in different genotypes. All strains were homozygous for the transgene Ppie-1mCherry::phPLCδ (ltIs44). nmy-2(S) 2nd, nmy-2(S-D) 2nd and nmy-2(S-A) 2nd represent the integrated single-copy transgenes Pnmy-2nmy-2 (bcSi96), Pnmy-2nmy-2S211DS1974D (bcSi103) and Pnmy-2nmy-2S211AS1974A (bcSi99), respectively. Each grey dot represents the ratio of one pair of daughter cells (n = 9–11). Red horizontal lines represent the mean ratio obtained for a given genotype, which is also shown on top. The black dotted horizontal line at a ratio of 0.64 represents the ratio of wild type. Statistical significance was determined using Mann–Whitney test (****, P≤0.0001, ns, no significance). (TIF) [file pgen.1008912.s008.tif]
