# Supplementary material for: PIG-1 MELK-dependent phosphorylation of nonmuscle myosin II promotes apoptosis through CES-1 Snail partitioning
Source: PLoS Genet. 2020 Sep 18;16(9):e1008912. doi: 10.1371/journal.pgen.1008912 (PMC7527206; doi:10.1371/journal.pgen.1008912)
Supplement: Attachment — Submitted filename: PLoS Genetics_Rebuttal_21July2020.docx [file pgen.1008912.s010.docx]

**Reviewer #1**

This manuscript describes a new role for the *C. elegans* maternal embryonic leucine zipper kinase orthologue *pig-1*, as well as its pathway components *mop-25.1/2*, *par-4* (LKB1) and *strd-1* (STRAD) in the asymmetric division and apoptotic death of Neuro-Secretory Motorneuron (NSM) sister cells. The demonstrate convincingly that *pig-1* is required for the polarized localization of nonmuscle myosin 2 NMY-2, and present genetic evidence that this pathway likely regulates the phosphorylation status and activity of NMY-2. Previously, Dr. Conradt’s lab showed that the SNAIL transcription factor CES-1 represses transcription of the pro-apoptotic BH3-only gene *egl-1* in NSMs. Here, they demonstrate that the *pig-1* pathway and NMY-2 function to maintain a gradient of CES-1 protein in the NSMs when they divide such that the smaller cell receives less CES-1 and therefore dies because it cannot repress *egl-1*. I found this to be a very solid study that makes an important advance in our understanding of how a cell of smaller size is specified to die after an asymmetric division. Below are a few comments for the authors to consider.

Why were *mop-25.1* and *-2* ablated together? I suspect there is redundancy, but this was not stated in the manuscript. This should be clarified in main text and data for single gene ablations included in the supplementary data section.

**Wei et al**

Denning et al (Nature 488:226, 2012) reported that *mop-25.1* and *mop-25.2* show functional redundancy. For this reason, we inactivated the genes individually and simultaneously. We did not see any phenotype with the single knock-downs in preliminary experiments and therefore, for subsequent experiments, focused on the double knock-down. We have included the following statement on page 8 of the ‘Result’ section:

*“(Denning et al., 2012 demonstrated that mop-25.1 and mop-25.2 are functionally redundant. For this reason, we inactivated the two genes simultaneously.)”* page 8

Figure 5A is a bit difficult to follow since there appears to be a lot of background fluorescence signal. I think a cartoon illustrating the distribution of the CES-1::mNeonGreen fusion protein in wild type versus *pig-1* mutants would be helpful to the reader.

**Wei et al**

We thank the reviewer for this suggestion. In **revised Fig. 7B** (Model figure), we are now providing a schematic of the CES-1 gradient not only in wild type but also in *pig-1* mutants. In addition, we have **revised Fig. 5A** and changed the order of the genotypes that we show. Specifically, we have moved the controls (*ces-2* and *ced-1*) to the bottom of the figure so that the images of the NSMnb in wild type are directly above the images of the NSMnb in *pig-1* mutants. We believe that this makes it easier to compare the two.

The authors claim that NMY-2::GFP levels are unchanged in *pig-1* mutant embryos (Fig S6), but the levels appear to be reduced in NSMnb (Fig 6A). Is it possible to accurately quantify total protein in this cell? If not, then it should be acknowledged in the discussion that an alternative interpretation would be that levels are altered specifically in the NSMs.

**Wei et al**

We have now also measured fluorescence intensity of NMY-2::GFP in the NSMnb (not only in the entire embryo) and found that there is a not significant decrease in the NSMnb in *pig-1* mutants (see **revised Fig. S6, new Part C**). We do not believe that this slight decrease can explain the lack of asymmetric enrichment observed in *pig-1* mutants, whose quantification we present in Fig. 6, Part B-D. However, it does suggest that *pig-1* might contribute to NMY-2 stability specifically in the NSM neuroblast lineage. Therefore, we have added the following statement in the Results section on page 14:

*“We also noted a slight, statistically not significant decrease in total fluorescence intensity of NMY-2::GFP in the NSMnb but not the entire embryo, which suggests that the loss of pig-1 may also affect the turn-over of NMY-2 protein in the NSM neuroblast lineage (Fig. S6).”*

Since the ts allele eliminates protein in all cells, it is possible that there is a cell non-autonomous role for NMY-2 in the asymmetric division and death of NSM sisters. Have the authors considered using the AID system to specifically deplete NMY-2 in the NSM cells to test autonomy? Alternatively, they could express the phosphomimetic *nmy-2* mutant transgene using the *tph-1* promoter to test for autonomy.

**Wei et al**

This is a great suggestion and we have considered this. Unfortunately, we are currently lacking a promoter that shows enough spatio-temporal specificity to perform this experiment (*tph-1* only starts to become expressed about 30 min after the division is completed).

The first sentence in the discussion is a bit of an over-statement, as it implies that MELK alters the phosphorylation of NMY-2 in the NSM lineage. This has not been shown biochemically (ie, using phospho-antibodies to NMY-2), so should be toned down. However, I think it would be unreasonable to request the authors to perform kinases assays to prove this point.

**Wei et al**

We agree with the reviewer and have inserted ‘directly or indirectly’ into this sentence, which now reads:

*“We present evidence in support of the notion that pig-1 MELK controls daughter cell size asymmetry and CES-1 Snail gradient formation in the NSM neuroblast lineage by directly or indirectly causing the phosphorylation at S211 and S1974 of nonmuscle myosin II NMY-2 (Figure 7A).”*

**Reviewer #2**

Asymmetric partitioning of cellular substances is an important mechanism for establishing cell asymmetry and determining cell fate. The C. elegans protein kinase PIG-1 is known to regulate asymmetrical cell division. This report further reveals the molecular mechanism PIG-1 utilizes to regulate downstream events, focusing on the regulation of apoptosis of the NSM sister cell. The authors found that CES-1, a critical repressor of the BH3-domain only protein and cell death inducer EGL-1, is asymmetrically distributed into the two daughter cells of NSMnb, one of which becomes NSM, the other undergoes apoptosis. Based on the genetic and cell biological study results reported here and on the previously published information about PIG-1, the authors propose a model that PIG-1 phosphorylates the non-muscle myosin molecule NMY-2 and this phosphorylation results in the asymmetric distribution of NMY-2 to the ventral side of the NSMnb. This event further facilitates the asymmetric division of the NSMnb into two daughter cells with different sizes, and the asymmetric distribution of CES-1; as a consequence, EGL-1 expressed is less repressed in the smaller sister cell and leads to the apoptosis of the smaller sister cell, whereas the larger sister cell becomes NSM. Furthermore, the authors showed that the C. elegans par-4, strd-1 or mop-25.1, 2 genes, which encode C. elegans homologs of LKB1, STRADα and MO25α, acts in the same pathway as pig-1 does, and propose that these genes act upstream of PIG-1 to activate it. In addition, integrating the authors’ previous finding, here the authors also propose an autoregulatory loop of CES-1 in which CES-1 inhibits the activity of PIG-1, which in turn regulates the asymmetric distribution of CES-1.


Significance

Although almost all apoptosis events are executed through a caspase-dependent pathway, there are many different upstream pathways that activate caspases in particular cells, in response to different signals. This report has discovered a specific apoptosis-initiation mechanism that acts through asymmetric distribution of a snail family transcription repressor that targets the expression of the cell death initiator EGL-1. This pathway is led by protein kinase PIG-1 and involves non-muscle myosin and thus probably the actomyosin network in generating a dorsal-ventral gradient of certain proteins inside a mother cell during cell division. This novel mechanism of apoptosis-initiation not only reveals the previously unknown roles of PIG-1 and the PAR-4 pathway proteins, but also broadens our knowledge regarding how many different ways apoptosis can be initiated.

Mammalian Maternal Embryonic Leucine zipper Kinase (MELK) protein is known to promote tumorigenesis, however, the mechanism behind this function remains unknown. On the other hand, C. elegans PIG-1, the ortholog of MELK, was reported to regulate asymmetrical cell fate in multiple lineages. The finding reported here will definitely shed light on our understanding of the function and regulation of mammalian MELK, and further on the mechanisms of tumorigenesis.

The auto-regulatory loop of CES-1 contributes to the precise control of CES-1 protein level in the three types of cells: NSMnb, NSM, and NSM sister cells. The discovery of this loop suggests that other snail family proteins might use the same regulatory mechanisms in cells.

This report thus will be of general interest to a broad audience in the research fields of cell biology, developmental biology, and cancer biology.

The experiments reported here are properly performed with the right controls and the results are rigorously analyzed, with repeats and quantifications. The conclusions are drawn based on the results and thus are solid. The models proposed here have integrated the findings made in this report and in the literature and thus are convincing. The report is clearly written. I enjoy reading this manuscript very much.


Specific points

A few points to be addressed are listed here. I do not intend to request new experiments. Addressing points 1 and 2 in Discussion, clarifying points 3 in the text, and revising point 4 would be sufficient.

1. The subcellular localization of CES-1::mNG (mNeonGreen).
Is CES-1 a nuclear protein? According to Fig. 5A, CES-1::mNG is in the cytoplasm or at least all over the cell even prior to metaphase. Is the asymmetrical partitioning of CES-1 coordinated with the nuclear envelope breakdown during mitosis? When does nuclear envelope breakdown occur in the context of CES-1 partitioning, which appears a rapidly dynamic event?

**Wei et al**

This is a very interesting point. We have not yet analysed NEBD in the NSM neuroblast lineage using a nuclear membrane marker. However, based on Nomarski optics, we believe that NEBD occurs as the NSMnb starts to divide. As noticed by the reviewer, we see CES-1 throughout the cell prior to that. Indeed, it is well documented that the activities of Snail-like transcription factors are controlled also at the level of nuclear import and export. Therefore, we speculate that CES-1 import in and export out of the nucleus might be controlled as well and that during the time frame that we observe the NSMnb, it is localized throughout the cell. To address the reviewers comment, we have added the following sentence in the Discussion section on pae 18:

*“How this is coordinated with other events that occur just prior to the actual NSMnb division, such as nuclear envelop breakdown, is currently unknown.”*

2. As the protein kinase that phosphorylates NMY-2, is PIG-1 also localized to the cell cortex and enriched on the ventral side in NSMnb like NMY-2? Knowing this information would potentially strengthen one part of the model proposed, that is, NMY-2 is a direct substrate of PIG-1.

**Wei et al**

We have obtained a fosmid-based multi-copy *pig-1::gfp* translational transgene and analysed its functionality and expression pattern. We found that the transgene is functional and we saw expression in actively dividing cells, which has previously been reported. However, we did not see any asymmetric localization or enrichment of PIG-1::GFP protein. To address the reviewers comment, we have added the following sentence to the Discussion section on page 17:

*“Whether PIG-1 protein itself is enriched in this part of the NSMnb is currently not known.”*

3. Fig. S4A was not mentioned in the text.

**Wei et al**

Fig. S4A is mentioned on page 11 of the Results section (line 8). It shows data confirming that the *ces-1::mNeonGreen* transgene is functional.

4. Fig. S5A: NMY-2::GFP time-lapse images are over-exposed, making it hard to judge the signal intensity?

**Wei et al**

We have **revised Fig. S5A** and reduced the brightness of all images to the same degree (images taken with identical exposure time).

**Reviewer #3**

MELK is over-expressed in some cancers, and shown to be required for clonogenic growth of breast cancer cells, although little was known about how it functioned. Pig-1, the C. elegans MELK ortholog, had previously been shown to regulate asymmetric cell division and apoptosis, providing an excellent model system to investigate its function further. This paper provides a thorough analysis of pig-1 in the NSM neuroblast, demonstrating a detailed pathway that may provide insight into MELK’s role in cancer. Overall, the findings are clearly presented, and novel tools are developed that provide new insight. However, many of these findings had previously been shown in other cell types, which reduces some of the novelty. For example, pig-1 had previously been shown to act in the same pathway as par-4, pig-1 had already been shown to affect non-muscle myosin localization, and pig-1 had already been shown to be a target of ces-1. Adjustments to the writing throughout the paper would help emphasize the new findings and indicate which results were consistent with previous findings. The new findings are that pig-1 leads to asymmetric localization of Ces-1 and thereby egl-1, and these are dependent on phosphorylation state of NMY-2. These are important findings that could explain resistance to apoptosis and cell fate changes observed in cancer cells.

Specific suggestions:
1. Fig. 2A and legend. It should be indicated that this is an infrequent event in pig-1 mutants. For example, 2% could be written on the side of the figure. As it is presented, it looks like all of the ‘NSM’ cells live longer.

**Wei et al**

As suggested by the reviewer, we have **revised Fig. 2A** and now indicate %NSMsc survival in wt and *pig-1* below the two different lineages.

2. What is the nature of the gm344 allele? Have other alleles been shown to behave similarly? It seems surprising that only one allele is investigated.

**Wei et al**

In a previous publication (Wei et al, Genetics 2017), we have demonstrated that in the NSM neuroblast lineage, two independent strong loss-of-function mutations of *pig-1*, *gm344* and *tm1510*, behave essentially identical with respect to NSMnb division and inappropriate NSMsc survival. For this reason, in the current study, we only used one of these mutations, *gm344*.

3. Fig. 4. It is noticeable that the level of egl-1 also becomes much higher in the pig-1 mutant, suggesting it takes more egl-1 to kill. This could be very interesting and could be mentioned.

**Wei et al**

We agree with the reviewer and have added the following statement on page 19 of the Discussion:

*“In the NSMsc, its larger cell size and increased amounts of CES-1 protein lead to a delay in egl-1 expression and cell death execution by about 10 min. In 98% of the cases, the cell still dies; however, the amount of EGL-1 that has accumulated in these cells at the time of death (based on reporter gene expression) is significantly higher compared to wild type, suggesting that cell death execution in these cells is compromised.”*

4. Egl-1 should be indicated in Fig. 7a.

**Wei et al**

As suggested by the reviewer, we have **revised Fig. 7A** and added *egl-1*.
